# Role of Technology Acceptance in the Telerehabilitation of Patients With Metabolic Syndrome: Longitudinal Study

**DOI:** 10.2196/82161

**Published:** 2026-05-14

**Authors:** Zsanett Tesch, Tamás Ujházi, István Kósa, Norbert Buzás

**Affiliations:** 1 Department of Health Economics Albert Szent-Györgyi Medical School University of Szeged Szeged Hungary; 2 Department of Preventive Medicine Albert Szent-Györgyi Medical School University of Szeged Szeged Hungary; 3 Department of Theoretical Health Sciences and Health Management Faculty of Health Sciences and Social Studies University of Szeged Szeged Hungary

**Keywords:** metabolic syndrome, modified UTAUT2, unified theory of acceptance and use of technology 2, patient behavior, technology acceptance, telerehabilitation

## Abstract

**Background:**

The advent of telerehabilitation has created new opportunities for the care of patients with metabolic syndrome. In distant rehabilitation, technology acceptance is particularly important because home-based projects are based on digital devices, and many patients are less familiar with their use.

**Objective:**

Our aim was to explore technology acceptance among patients undergoing a 3-month complex, telemedicine-supported metabolic rehabilitation. We were curious to see how different factors influence the intention to use rehabilitation technologies and how this changes through the telerehabilitation process.

**Methods:**

Participants were selected from patients in the metabolic telerehabilitation program at the university. Our model was based on the unified theory of acceptance and use of technology 2, which we supplemented with various other constructs. A paper-pencil questionnaire survey was administered on the last day of the preparatory week of the rehabilitation program (T1, n=145) and at the follow-up visit after the closing (T2, n=139). We used structural equation modeling with the least squares method to explore the relationships between model variables. Respondent segments were also identified by performing a hierarchical cluster analysis using Ward’s method.

**Results:**

Facilitating conditions (FC) have the greatest impact (0.366) on the behavioral intention (BI) to use technology. Effort expectancy has no direct effect on BI; it operates only through performance expectancy (PE), which may be because, in telerehabilitation settings, patients are more goal-driven than experience-driven. The analyses of the T2 data show that the direct impact of social influence on BI has disappeared by the end of the rehabilitation process. This can be explained by the fact that during device use, it becomes clear that the devices are secure and the data are safe, making this factor implicit in the patient’s behavior. Only 2 constructs appeared in both the T1 and T2 models: PE and FC. By comparing the 2 datasets, we have provided empirical support for an old hypothesis: the experience of using the tool for a time has led to a significant reduction in the impact of FC and a corresponding increase in the dominance of PE, which has “absorbed” the impact of some other constructs. Based on respondents’ attitudes, we found 3 clusters. The telerehabilitation program itself has a significant impact on patients’ BI, as the relative share of “enthusiastic users” (73/145, 50.3%) increased by about 20%, while the share of “distrustful reluctants” (25/145, 17.2%) decreased to a quarter by the end of the program.

**Conclusions:**

This behavior-based functional approach enables treatments to be tailored to actual technology-use demands rather than to presumptive societal features. This means that before beginning rehabilitation, attempts should be undertaken to identify patients’ clusters in clinical practice, and rehabilitation should be planned according to the individual’s attitude toward technology.

## Introduction

### Background

Obesity and metabolic syndrome, as a consequence, are serious public health challenges worldwide, significantly raising the risk of cardiovascular disease, type 2 diabetes, and other chronic conditions [1,2]. The prevalence of obesity has increased steadily in recent decades, not only in affluent countries but also globally, including in middle- and low-income regions [3]. Structured rehabilitation programs play a key role in the care of patients with metabolic syndrome, but access to these programs is limited. Traditional, location-based rehabilitation programs often face capacity constraints, long waiting lists, and geographical or mobility limitations of patients [4-7]. Telerehabilitation offers an alternative to these problems, enabling the therapeutic process to be carried out in the home environment using mobile technology and digital platforms [5].

The 2 most common uses of telerehabilitation are cardiac and poststroke rehabilitation. Mobile technology has been well accepted in rehabilitation programs for patients with ischemic heart disease and congestive heart failure, and digital self-confidence was a significant predictor of acceptance [8]. A comparison of a technology-based, home-based, remotely monitored program and a traditional cardiac rehabilitation program showed that the home program is feasible and acceptable to patients, but that home rehabilitation presents challenges, including maintaining motivation over the long term, which makes its widespread implementation difficult [9]. A focus group study of 188 patients examining telemedicine rehabilitation care for people with chronic diseases [10] found that the possibility of exercising at home, working on one’s own recovery, and receiving better-quality exercise instruction thanks to exercise videos on the portal were among the factors that facilitated the acceptance of mobile health (mHealth) rehabilitation. The majority of participants also viewed the benefits of the telerehabilitation program for survivors of stroke as positive [11]. Widespread mobile phone use has helped to make rehabilitation strategies involving the families of survivors of stroke and remote monitoring via telemedicine feasible and cost-effective even in low- and middle-income countries. Participants in another stroke telerehabilitation program also rated regular contact positively, particularly the provision of care-related information and exercise reminders, which helped them to maintain their commitment to the exercise program [12].

Telemedicine has become a key tool in digital health care over the past decade, with the COVID-19 pandemic accelerating the adoption of digital solutions [13]. However, the success of telemedicine-supported programs varies a lot around the world. The main barriers are technology-specific [14], as the success of a program depends on its user-friendly interface and ease of use, especially for older or less technologically experienced users [15]. On the other hand, the effectiveness of programs strongly depends on patients’ general attitudes toward technology use, digital competence, socialization background, and perceived privacy in their personal sphere [16,17].

### Prior Work

The basis for predicting technology acceptance behaviors can be traced back to the theory of reasoned action of Fishbein and Ajzen [18], which posits that the adoption of a given behavior depends on an individual’s attitude and subjective norms. Davis [19] further developed this framework specifically for the study of technology acceptance, creating the technology acceptance model (TAM), in which perceived usefulness and perceived ease of use determine the intention to use. As a next development of the model, Venkatesh and Davis [20] identified factors influencing perceived usefulness in the TAM 2 model, followed by Venkatesh and Bala [21] identifying factors influencing perceived ease of use in the TAM 3 model. The consecutive step was the unified theory of acceptance and use of technology (UTAUT), developed by Venkatesh et al [22]. A further development of this, the unified theory of acceptance and use of technology 2 (UTAUT2) model [23], specifically examines technology adoption among everyday consumers. The research frameworks for exploring technology acceptance listed here share the commonality that they examine use intention and actual use as target variables. Intent to use reflects the extent to which consumers are open to adopting a new technology based on their existing attitudes, while actual use reveals how much time they spend using the device once they have the opportunity.

In their comprehensive methodological study, Holden and Karsh [24] pointed out more than a decade ago that an important future direction for technology acceptance is to adapt the model explicitly to the health context. Notwithstanding, a decade later, Niknejad et al [25] still identified in telerehabilitation far fewer studies than expected that had shown a quantitative association between promoting factors or inhibitors of telerehabilitation adoption and intention to use. Patient-side adoption of telemedicine is influenced by a number of factors that are not part of classical models explaining technology acceptance but are more specific, contextual determinants [10]. Liu et al [26] showed that expected performance was the strongest predictor of intention to use technology for rehabilitation, whereas effort expectancy (EE) and social impact were not significant. In contrast, facilitating conditions (FC) significantly influenced not only intention but also the actual use. Koo et al [27], analyzing the adoption of personalized health management mobile apps by older patients, highlighted the role of performance expectancy (PE), EE, FC, social influence (SI), habit, hedonic motivation (HM), and trust. Huang et al [28] also confirmed the influence of TAM variables for electronic health recording and sharing apps, while Fezzi et al [29] identified perceived usefulness, perceived ease of use, skill level, task complexity, and the nature of the technology as influencing factors for functional insulin therapy. Cooks et al [30] associated the acceptance of virtual health care professionals (HPs) with a sense of belonging, local identity, self-concept, similarity, and attractiveness, while Kauttonen et al [31] found that the acceptance of artificial intelligence–based health care apps is determined by trust, data security, and the role of the vendor. Schomakers et al [32] and Siebert et al [33] noted that, for therapeutic mobile apps, trust is an independent explanatory factor alongside the UTAUT construct. Rajak and Shaw [16] attempted to generalize the TAM model to mHealth users and demonstrated its high predictive power. Zhu et al [34] used the UTAUT2 model for a similar purpose and demonstrated that the model’s original constructs have a meaningful impact on the intention to use. Schretzlmaier et al [35] also extended the UTAUT2 model with their own constructs and found that the factors trust and received threat of illness are also relevant for the acceptance of mHealth technologies. Yang et al [17], building on the UTAUT theory, have recently demonstrated the impact of the convenience, accuracy, and privacy protection factors on technology intention to use. Examining the adoption of mHealth technologies in a national sample, it was found that, in addition to the classic variables of the UTAUT2 model, privacy, lifestyle, self-efficacy, and trust in Bangladesh [36], while resistance to use, data privacy issues, and HM in the Philippines [37], also influence behavioral intention (BI) to use.

In conclusion, in addition to the application of classical TAMs such as TAM and UTAUT, there is a growing emphasis in practice on the inclusion of psychological and technology-specific constructs that make the explanation of technology acceptance in health care more complex and viable. It is also evident from decades of practice in the widespread use of models that constructs influencing target variables can be freely modified by dropping some constructs or adding new ones to suit specific research contexts or objectives [38]. In line with this, AlQudah et al [39] also emphasize the scope to integrate new constructs to enhance the robustness of models of user acceptance of new technologies in health care. This is also confirmed by Ramachandran et al [40], who argue that although the usefulness of home-based telemedicine-supported rehabilitation programs is clear to patients, adoption is also influenced by a number of external factors.

### Study Objectives and Proposed Hypotheses

#### Overview

The aim of this study is to investigate the factors influencing technology acceptance in a telemedicine-assisted rehabilitation program for patients with metabolic syndrome. Our aim was to investigate how constructs influence the intention to use technology and, in turn, to develop an acceptance model for the telerehabilitation process for patients with metabolic syndrome. On the other hand, we sought to answer how the experience of the telerehabilitation process influences patients’ technology acceptance parameters, that is, how participation in the process shapes their intention to use.

Our modeling was based on the UTAUT2 model [23]. Since the technologies provided to users were free, we omitted the price construct from our model. We did the same with habit, as the participants in the rehabilitation program did not have a routine in using the devices. For the remaining constructs in the UTAUT2 model, we assumed the specified impacts [23] on the intention to use rehabilitation technologies (hypothesis 1).

**Figure 1:**
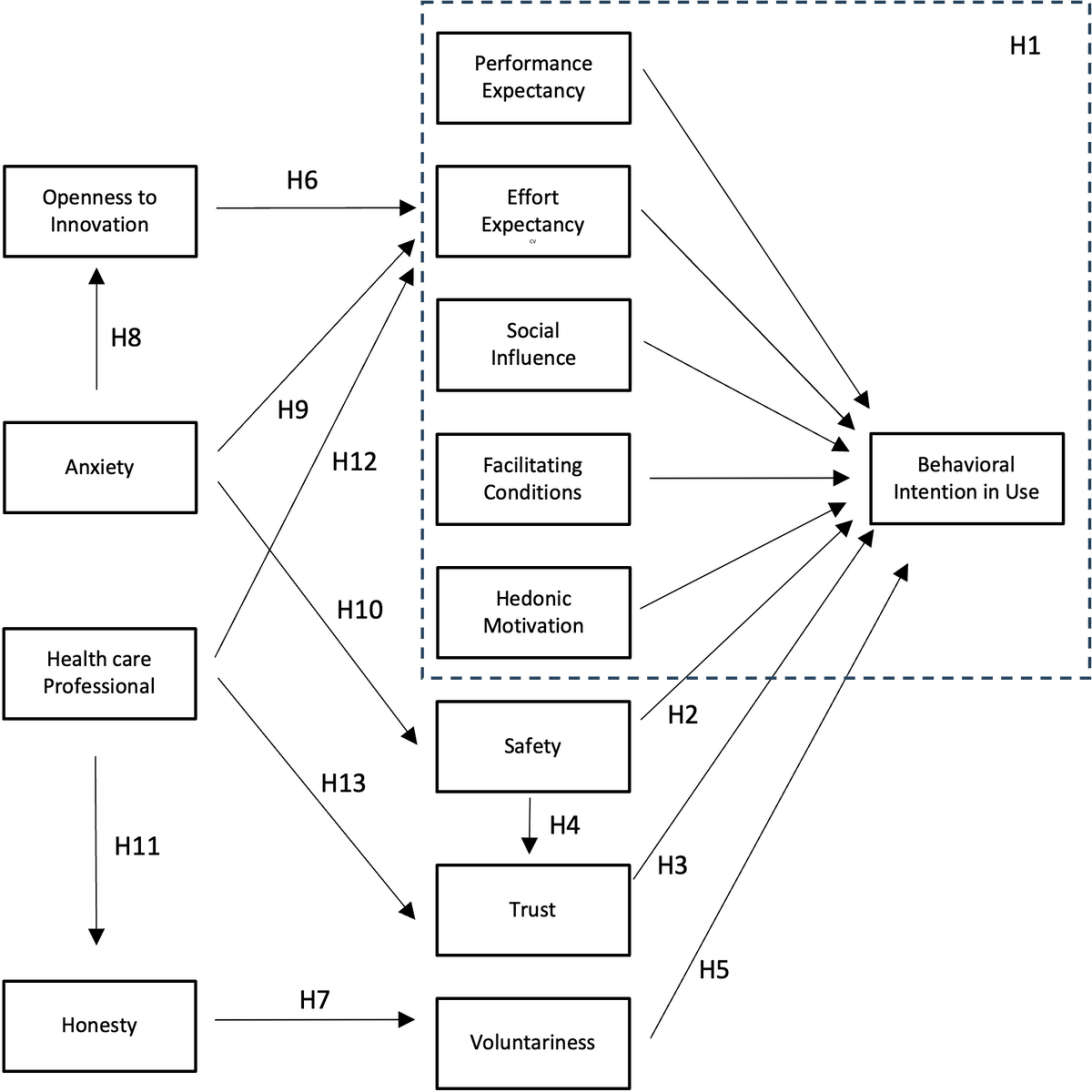
The extended UTAUT2 model as research hypotheses. H: hypothesis; UTAUT2: unified theory of acceptance and use of technology 2.

To further describe the factors influencing technology adoption in metabolic rehabilitation, we also conducted an exploratory qualitative study. The aim of this was to identify additional relevant constructs that may influence the intention to use the technologies, based on the experiences of professionals directly involved in the program, in line with the suggestion of Kapser and Abdelrahman [38], and to incorporate them into our baseline model. Data collection consisted of 1-hour semistructured interviews with a total of 3 physicians, 2 nurses, 2 dietitians, and 2 physiotherapists from different specialized groups within the clinic. The participants all had direct contact with patients undergoing metabolic rehabilitation and were thus able to provide meaningful insights into the manifestation of attitudes, barriers, and supportive factors related to technology use. The information gathered was subjected to a content analysis, which revealed several factors that are less or differently emphasized in models in the literature. Therefore, we have included these constructs (safety, trust, voluntariness, openness to innovation [OP], honesty, anxiety, and HPs) in our conceptual model (Figure 1), and we explain their use in detail below.

#### Safety

A sense of safety plays a key role in the acceptance of health technologies, especially for patients who use digital or invasive devices regularly. In this context, the safety factor refers to the extent to which the user perceives the technology as physically harmless, psychologically reassuring, technologically reliable, and privacy-safe [41]. Kitsiou et al [42] also showed that perceptions of safety have a significant positive impact on intention to use, especially in sensitive application areas such as continuous glucose monitoring or remote monitoring systems. Examining the use of home telehealth services among older people, Cimperman et al [43] also found that perceived safety positively influences the intention to use. Lack of safety (eg, fear of device malfunction or data leakage), which is effectively equivalent to the perceived threat variable [34,44], can significantly reduce trust in technology, which plays a key mediating role in acceptance models. On this basis, we hypothesized that the safety construct would positively affect the BI to use rehabilitation technologies (hypothesis 2).

#### Trust

Trust plays a key role in technology adoption, as patients are in a vulnerable position in health care. Trust, in this context, reflects the user’s belief that the technology is reliable, predictable, and safe to use during the treatment process [45]. In the case of telemedicine applications, this belief extends to the system’s technical stability, data security, and the belief that HPs will use the device appropriately and ethically [46]. Higher levels of trust can significantly increase the intention to use the technology [34-36,47]. Accordingly, in our modeling, we hypothesized that trust would have a positive effect on intention to use for rehabilitation technologies (hypothesis 3). However, we also hypothesized that safety would have a positive effect on trust, also because safety enhances overall trust (hypothesis 4).

#### Voluntariness

Voluntariness is a key factor in technology adoption models, especially in environments where users are sensitive to issues affecting their autonomy, such as health care. This latent variable measures the extent to which a user feels that the use of technology is based on free choice rather than on external constraints or expectations [22]. A sense of voluntariness contributes significantly to the development of positive attitudes, increases willingness to accept, and reduces resistance, especially when new technologies (eg, telehealth systems or wearable sensors) are introduced to populations vulnerable to a loss of control [48]. In a clinical setting, it is particularly important that patients perceive technology as a choice rather than an instruction from the treating physician or institution, as this indirectly influences the likelihood of engagement and long-term use. Voluntariness, as a latent variable, appears as a moderating variable in the UTAUT1 model but is no longer included in the UTAUT2 model [23], as it is implicit in the consumer context that consumption is voluntary. Since in health care, a patient may always feel “pressure” from health care staff, so voluntariness is not implicit, we reintegrated this variable into the model and assumed that voluntariness positively influences intention to use rehabilitation technologies (hypothesis 5).

#### Openness to Innovation

OP reflects an individual’s willingness and ability to try new technologies, solutions, or services, even if they initially have little information about how they work. This personality dimension often serves as a moderator or indirect variable in models of technology adoption, as it significantly impacts attitudes toward technological innovations and, in turn, the intention to use them [49]. Individuals with high OP tend to be more willing to use digital health solutions, such as telemedicine applications, and are less likely to be apprehensive about novelty, making it easier for them to turn initial experimentation into regular use [50]. In the context of metabolic rehabilitation, this is particularly relevant, as patients need to regularly monitor their own condition and provide data, which requires the use of digital tools, the acceptance of which can be greatly facilitated by a positive attitude toward technological novelty. As openness is primarily associated with technology-use efforts, we hypothesized that OP would have a positive effect on EE within the rehabilitation technology adoption model (hypothesis 6).

#### Honesty

Based on the experience of qualitative in-depth interviews with health care staff, we included social effects such as honesty. This is the willingness of users to share sensitive personal information when using the tools provided to them [51]. This depends on the provider’s transparent data management and open communication with the user [52]. These suggest that user honesty, as a specific form of social interaction, can be incorporated into the TAM in digital tool–assisted rehabilitation, where accurate, fair, and voluntary data disclosure is key to effectiveness (hypothesis 7).

#### Anxiety

Anxiety describes the negative anticipatory reactions and emotional responses associated with technology use, arising from fear of use, fear of making mistakes, or experiencing the complexity and unpredictability of technology. This psychological factor is particularly important in digital health settings, where users often perceive improper use as a health risk [53]. Technology anxiety often negatively impacts perceived ease of use and perceived usefulness, thereby weakening the intention to use [54]. This is particularly relevant for patients undergoing metabolic rehabilitation, as most have limited digital experience and may be more sensitive to the risk of digital system failure due to their health conditions. Anxiety may therefore also be a barrier to OP (hypothesis 8), EE (hypothesis 9), and sense of safety (hypothesis 10).

#### Health Care Professionals

From the construct called SI in previous models, we have separated the influence of HPs to more accurately describe the effects, as their role is multifaceted and more actively helping than the encouraging role of family or friends. In particular, doctors, nurses, physiotherapists, and dieticians have a significant impact on patients’ willingness to use technology in rehabilitation when they introduce new digital solutions such as telemedicine systems or mHealth apps. For patients, health care staff are a key source of information and a point of trust, so their positive attitudes may facilitate technology acceptance, while their skepticism or passivity may prevent it [55]. Accordingly, we hypothesized that HP would positively impact the honesty (hypothesis 11), EE (hypothesis 12), and trust (hypothesis 13) factors.

## Methods

### Sample Size Estimation

Given the finite population of eligible program participants (N=170), we calculated the minimum required sample size using a conservative proportion-based approach (*P*=.50), a 95% confidence level, and a 5% margin of error with a finite population correction, resulting in a minimum of 118 participants. Our achieved sample size exceeded this threshold (T1: n=145; T2: n=139). We also included a power-based verification aligned with the primary partial least squares-structural equation modeling model, confirming that the baseline sample provides adequate power (α=.05; 80% power) to predict BI from the number of model predictors.

### Recruitment

Participants in the study were selected from patients in the metabolic telerehabilitation program at the Cardiometabolic Rehabilitation Unit of the Internal Medicine Clinic, University of Szeged, using convenience sampling. The study included individuals between the ages of 25 and 70 years who had low levels of physical activity (less than 30 minutes of self-reported exercise per week) and lived with metabolic syndrome. To define metabolic syndrome, we used the Adult Treatment Panel-III criteria [56], according to which, participants must have at least 3 of the 5 risk factors listed therein, as determined from a review of their medical records.

Individuals with any of the following conditions were excluded from selection: planned invasive cardiological intervention, uncontrolled hypertension, type 1 diabetes mellitus, type 2 diabetes treated with more than 1 daily dose of insulin, heart failure, renal failure, cancer, severe cognitive impairment, lack of cooperation, known conditions preventing physical training, and mental conditions severely limiting judgment or capacity to act.

### Questionnaire Development

To measure the variables in the model shown in Figure 1, a self-completion questionnaire was developed through a multistep procedure to ensure adequate content and face validity. The selection of constructs was grounded in the theoretical framework and the qualitative semistructured interviews described earlier. Content validity was ensured by aligning questionnaire items with both established literature-based measurement scales and the expert-derived thematic findings. The preliminary questionnaire was reviewed by members of the research team with expertise in health economics, preventive medicine, and digital health research to evaluate item relevance, clarity, and domain coverage. Face validity was assessed through a pilot evaluation involving representatives of the target population participating in the rehabilitation preparation phase, focusing on the comprehensibility, clarity of wording, and interpretability of the items. Based on feedback received, minor linguistic and formatting adjustments were implemented before administering the final questionnaire. All items were measured using a 5-point Likert scale, reflecting the level of agreement with each statement. The constructs and corresponding measurement items are presented in Table 1.

The translation of the questions was carried out using the following method: 2 independent experts translated them, and their translations were then reconciled. The reconciled version was back-translated and approved by the corresponding author (NB).

**Table 1:** Variables and measurement items in the UTAUT2^a^ model for telerehabilitation.

Constructs and items		Content		Loadings		Cronbach α		CR^b^		AVE^c^
**Anxiety (AX)**						0.932		0.950		0.879
	AX1	Nervousness	0.945							
	AX2	Confusion	0.945							
	AX3	Uncomfortability	0.922							
**Behavioral intention (BI)**						0.921		0.924		0.864
	BI1	Intention	0.934							
	BI2	Frequency	0.954							
	BI3	Continuity	0.899							
**Effort expectancy (EE)**						0.913		0.937		0.796
	EE1	Easy to learn	0.919							
	EE2	User-friendliness	0.944							
	EE3	Easy to use	0.928							
	EE4	No special skills needed	0.767							
**Facilitating conditions (FC)**						0.858		0.868		0.701
	FC1	Equipment conditions	0.847							
	FC2	Troubleshooting	0.871							
	FC3	Compatibility	0.791							
	FC4	Technical support	0.838							
**Hedonic motivation (HM)**						0.873		0.969		0.792
	HM1	Fun	0.871							
	HM2	Joy	0.94							
	HM3	Entertainment	0.857							
**Health care professionals (HPs)**						0.908		0.882		0.787
	HP1	Doctors	0.896							
	HP2	Dietitians	0.843							
	HP3	Physiotherapists	0.925							
	HP4	Nurses	0.876							
**Honesty (HO)**						0.744		0.911		0.784
	HO1	True data	0.942							
	HO2	Embarrassment	0.828							
**Openness to innovation (OP)**						0.815		0.868		0.722
	OP1	Openness to new products	0.875							
	OP2	Up-to-date in technology	0.875							
	OP3	First user of a new technology	0.797							
**Performance expectancy (PE)**						0.853		0.867		0.695
	PE1	Usefulness	0.784							
	PE2	Accomplishments	0.891							
	PE3	Productivity	0.872							
	PE4	Data tracking	0.782							
**Safety (SA)**						0.8		0.798		0.718
	SA1	Security	0.75							
	SA2	Safety of data	0.905							
	SA3	Confidentiality	0.879							
**Social influence (SI)**						0.789		0.797		0.704
	SI1	Influence of close people	0.873							
	SI2	Proudness	0.794							
	SI3	Friends’ encouragement	0.847							
**Trust (TR)**						0.863		0.874		0.708
	TR1	Trustworthiness	0.806							
	TR2	Privacy	0.868							
	TR3	Reliability	0.845							
	TR4	Safety of device	0.845							
**Voluntariness (VO)**										
	VO1	Free will	1		—^d^		—		—	

^a^UTAUT2: unified theory of acceptance and use of technology 2.

^b^CR: composite reliability.

^c^AVE: average variance extracted.

^d^Not applicable.

### Data Collection

In this study, a paper-pencil questionnaire was administered to patients with metabolic syndrome who had participated in a 3-month complex rehabilitation program on 2 occasions: on the last day of the preparatory week prior to the rehabilitation program (T1) and at the follow-up visit after the program closed (T2). Patients completed the questionnaires in a separate, quiet room. All questionnaires were recorded in an electronic database by one of the researchers (ZT).

### Statistical Analysis

The first step in analyzing the collected data was to use descriptive statistics to describe the sample population’s sociodemographic characteristics. To answer our research question, we used structural equation modeling with the least squares method to explore the relationships among model variables using SmartPLS software (version 4; SmartPLS GmbH). The path analysis allowed us to investigate the extent to which each variable directly influences the intention to use the devices dispensed to patients during metabolic telerehabilitation and to identify interactions between these variables, thereby identifying which variables have an indirect effect on intention to use. Finally, to provide a deeper interpretation of the results, respondent segments were identified through a hierarchical cluster analysis using Ward’s method. The procedure uses the participants’ responses to the indicators as criteria and, based on these, classifies those with similar responses into similar inwardly homogeneous clusters, while the system ensures that the different clusters are heterogeneous along their criteria, that is, well distinguishable. The output of the cluster analysis is to identify clusters by saving the clusters as variables and comparing averages along the indicator variables to identify the respondent groups.

### Ethical Considerations

The study was conducted in accordance with the guidelines of the Declaration of Helsinki and was approved by the Medical Research Council of Hungary (approval BM/25016-1/2024). The research was based on prior written informed consent from the participants. The questionnaire was completed in a separate room, with participants seated at a distance from one another to ensure privacy. After completing the questionnaire, participants could not be identified, thus ensuring confidentiality. Participants did not receive any compensation for their participation in the research.

## Results

### Demographic Characteristics

The demographic profile of respondents (n=145) is shown in Table 2. During the study period, 58.5% (83/145) of the participants in the telemedicine-supported metabolic rehabilitation program were female, and 41.5% (59/145) were male. Patients aged 25 to 82 years (mean age 52.6, SD 12.65 years) completed the questionnaire. In terms of age distribution, 19.6% (28/145) were younger than 40 years, almost half were between 51 and 70 years, and only 9.8% (14/145) were older than 70 years. In total, 28.7% (41/145) of respondents came from a large city, 37.8% (54/145) from a small town, and 1 in 3 from a village. In terms of education, nearly two-thirds of respondents had a secondary education, one-third had a higher education degree, and 2.1% (3/145) had only a primary education. In terms of marital status, 63.9% of respondents are in a couple, 43.7% have children, and 29.9% declared themselves completely single. Regarding their financial means, 45.5% (66/145) of respondents said that everyday expenses are not a problem, that they can afford some extra expenses in their monthly budget, but that they cannot save. A total of 36.6% (53/145) of respondents can afford extra spending beyond living expenses and can save at the end of the month. Only 2.8% (4/145) of respondents have serious financial difficulties.

**Table 2:** Participants’ characteristics.

Demographic factors				Values, n (%)
**Sex**				
	Male	59 (41.5)		
	Female	83 (58.5)		
**Age (years)**				
	≤40	28 (19.6)		
	41-50	31 (21.7)		
	51-60	36 (25.2)		
	61-70	34 (23.8)		
	≥70	14 (9.8)		
**Marital status**				
	Married or relationship without children	38 (26.4)		
	Married or relationship with children	54 (37.5)		
	Single without children	43 (29.9)		
	Single with children	9 (6.3)		
**Place of residence**				
	Bigger city	41 (28.7)		
	Small city	54 (37.8)		
	Village	48 (33.6)		
**Highest level of education**				
	Primary school		3 (2.1)	
	Vocational college		45 (31)	
	Secondary school		46 (31.7)	
	College or university		47 (32.4)	
	Other		4 (2.8)	
**Financial situation**				
	My monthly income is not enough to buy basic necessities		4 (2.8)	
	I can buy basic necessities, but I have no money for anything else; it just about lasts until the end of the month		21 (14.5)	
	I can buy basic necessities and occasionally allow myself 1 or 2 extra expenses, but I cannot save any money		66 (45.5)	
	I can buy what I need and even put some money aside, and I can afford occasional extra expenses		53 (36.6)	
	I can buy what I need and even put some money aside, and I can afford regular extra expenses		1 (0.7)	

### Partial Least Squares-Structural Equation Modeling Model

The results confirming the reliability and validity of the constructs are also summarized in Table 1. Construct reliability was assessed using the Cronbach α coefficient and the composite reliability (CR) coefficient [57]. The CR is more accurate because it accounts for the model structure and covariances. Both coefficients above 0.70 are considered satisfactory. The results showed that Cronbach α and CR were all above 0.7, with the lowest values being 0.744 and 0.797, respectively. However, none of the values exceeded the redundancy threshold of 0.95, indicating that the constructs are reliable. The convergent validity of the constructs was determined using average variance extracted. The results showed that all average variance extracted values were above 0.5, indicating adequate convergent validity for all constructs, reflecting their 1D nature.

**Figure 2:**
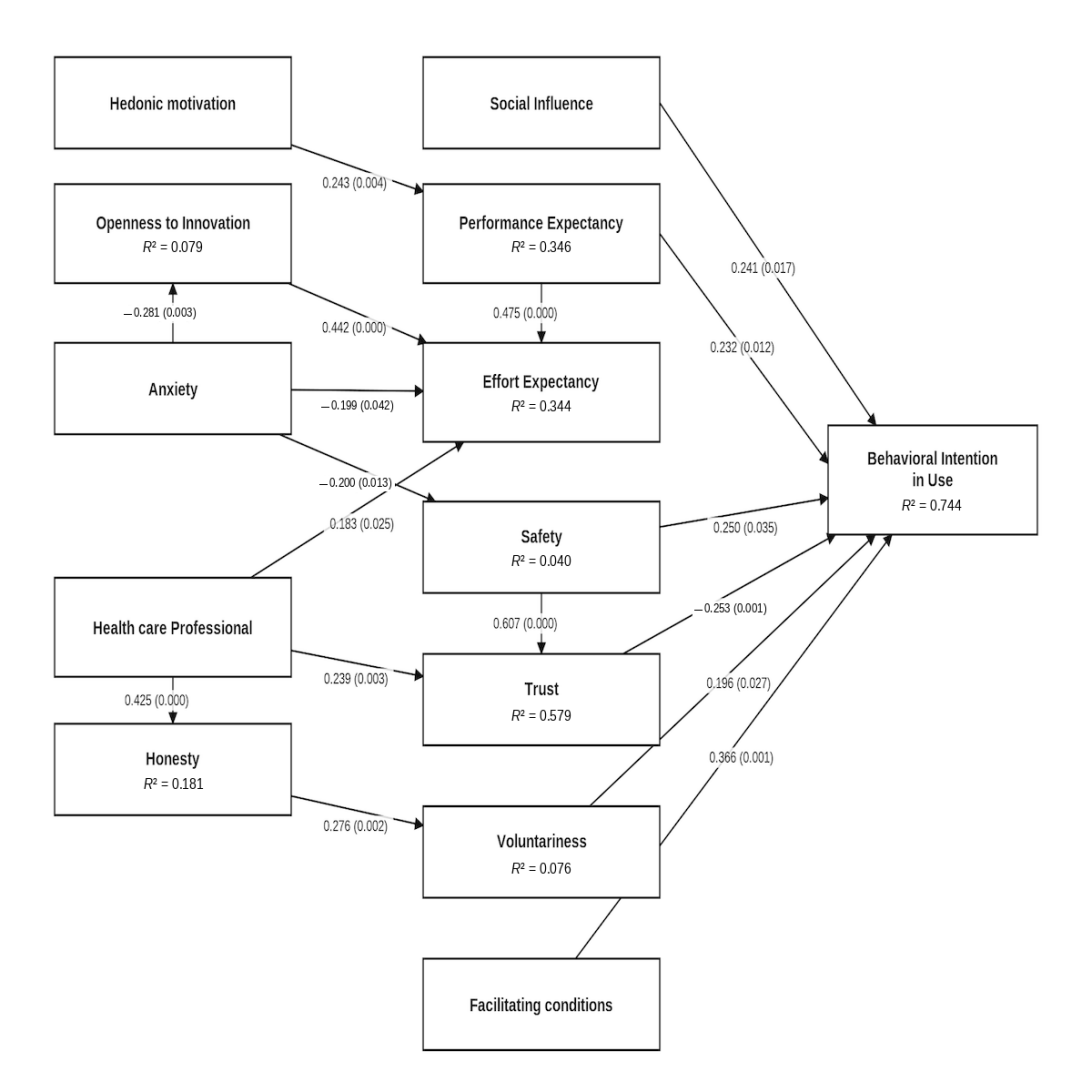
Extended UTAUT2 model with latent variables and path coefficients in T1. UTAUT2: unified theory of acceptance and use of technology 2.

Table 3 presents the hypothesis testing results for the path analysis based on the questionnaires collected in T1, and Figure 2 shows their graphical representation. Table S1 in Multimedia Appendix 1 contains the more detailed statistical parameters. It can be seen that the intention to use (BI) is directly and positively influenced by the PE, SI, and FC factors, all of which are included in the UTAUT2 model. No direct effect of EE was detected, and HM only has an indirect effect on intention to use through PE. Thus, we have only partially confirmed hypothesis 1.

**Table 3:** Hypothesis testing summary for T1 and T2 surveys.

Path	Hypothesis (H)	T1			T2	
		Original sample	*P* value	Original sample		*P* value
PE^a^→BI^b^	H1	0.232	.01	0.403		<.001
EE^c^→BI	H1	Not confirmed	—^d^	Not confirmed		—
SI^e^→BI	H1	0.241	.02	Not confirmed		—
FC^f^→BI	H1	0.366	.001	0.266		.01
HM^g^→BI	H1	Not confirmed	—	Not confirmed		—
EE→PE	Not presumed	0.475	<.001	0.315		.02
HM→PE	Not presumed	0.243	.004	0.334		.003
SA^h^→BI	H2	0.25	.04	Not confirmed		—
TR^i^→BI	H3	–0.253	.001	Not confirmed		—
SA→TR	H4	0.607	<.001	0.411		<.001
VO^j^→BI	H5	0.196	.03	Not confirmed		—
OP^k^→EE	H6	0.442	<.001	Not confirmed		—
HO^l^→VO	H7	0.276	.002	Not confirmed		—
AX^m^→OP	H8	–0.281	.003	–0.416		<.001
AX→EE	H9	–0.199	.04	–0.53		<.001
AX→SA	H10	–0.2	.01	–0.462		<.001
HP^n^→HO	H11	0.425	<.001	Not confirmed		—
HP→EE	H12	0.183	.03	0.283		.001
HP→TR	H13	0.239	.003	0.268		.004

^a^PE: performance expectancy.

^b^BI: behavioral intention.

^c^EE: effort expectancy.

^d^Not available.

^e^SI: social influence.

^f^FC: facilitating condition.

^g^HM: hedonic motivation.

^h^SA: safety.

^i^TR: trust.

^j^VO: voluntariness.

^k^OP: openness to innovation.

^l^HO: honesty.

^m^AX: anxiety.

^n^HP: health care professional.

In addition to the above, BI is also directly positively affected by safety and voluntariness. Therefore, hypotheses 2 and 5 are accepted. Trust also has a direct negative effect on BI, so hypothesis 3 is not confirmed. Safety, on the other hand, has a very strong effect (0.607) on trust, confirming hypothesis 4. Although EE does not have a direct effect on BI, it has a strong effect on PE (0.475), indicating that the tools used in the telerehabilitation program are easy and user-friendly without prior training.

In terms of the strength of constructs directly affecting intention to use, the largest influence (0.366) is FC, which includes 4 items: the conditions in the rehabilitation participant’s home, the problem-solving ability required to use rehabilitation tools, the compatibility of the tools used with those the user is currently using, and the expected assistance with problems that would arise during use. Nearly equally influential on BI is PE (0.232), that is, the perception of how useful the rehabilitation program and the tools used in it will be in achieving the goals set. Just like SI is 0.241, which indicates the influence of close people to the patient; and safety is 0.250, which indicates that the perception of security in the use of the devices and the inaccessibility of the data generated by the use of the devices to unauthorized persons. Voluntariness, that is, the intention to use the system despite the fact that its use is not compulsory, is less significant (0.196) than the former ones.

Examining which other constructs moderate the direct influence on intention to use, we see that FC and SI are not related to other factors. Trust is significantly influenced by HP (0.239), that is, the expectation of active assistance from health staff is a clear contributor to the perceived safety. HP also has a significant effect on honesty (0.425), implying that the attitude of doctors, nurses, dieticians, and physiotherapists toward patients contributes to the honesty of the data. HM, which has a direct effect on BI in the UTAUT2 model, has a direct effect on PE here, indicating that performance expectations are also proportional to the enjoyment of technology. Anxiety, as originally hypothesized, also affects 3 other factors: OP, EE, and safety. The effect is negative in all 3 cases: lower innovation anxiety is beneficial for openness to new technologies, expectations of the effort required to operate, and perceptions of safety. Figure 2 also shows that all hypotheses (hypotheses 6-13) related to constructs with a moderating effect were confirmed.

To investigate how participation in the rehabilitation program influences intention to use technology, we also conducted a survey in T2 among patients (n=139) returning to the final medical control at the end of the program. The results of this path analysis are also shown in Table 3. Additional statistical parameters are provided in Table S2 in Multimedia Appendix 1. The model appears to have been simplified. The effect of SI on intention to use was eliminated, so the variable was eliminated from the model. In addition, the effects of voluntariness on BI and of honesty on voluntariness have been discontinued, so voluntariness has disappeared from the model. The direct effects of trust and safety on BI have also ceased, so that only FC and PE have an effect on the intention to use after the rehabilitation program. The effect relationships of anxiety and their sign have not changed, but their effects have increased significantly. The effects of HP also remained unchanged, but while its influence on honesty was weakened, its influence on EE was significantly strengthened.

### Cluster Analysis

#### Overview

TAMs provide a useful framework for understanding how patients perceive digital health solutions in the context of telerehabilitation. However, these models assume linear relationships and cannot capture heterogeneity within patient populations or the diverse patterns of technology use. Based on this consideration, we also apply cluster analysis to our research to identify distinct groups of patients with metabolic syndrome characterized by different attitudes and behavior patterns.

By performing hierarchical cluster analysis on the questionnaire data recorded at T1 (using a dendrogram created with a combination of Ward’s minimum variance clustering algorithm and squared Euclidean distance), 3 distinct clusters were identified. The groups are heterogeneous among themselves and homogeneous within the cluster. The clusters are not segregated by demographic characteristics; no category of age, education, residence, or income is predominant in any cluster. Consequently, clusters were further analyzed by comparing the means of responses to questions across variables, that is, functional behavioral patterns, attitudes, emotional reactions, and social support related to digital technology use.

Based on the attitudes of the respondents, the 3 distinct groups and their characteristics are described.

#### Enthusiastic Users

About half of the participants (73/145, 50.3%) belong to this group. They are open and active in their attitudes toward the use of technology. They enjoy using rehabilitation tools and are eager to try new technologies. Their characteristics are as follows: they most enjoy using rehabilitation tools, they are most likely to try new technologies, and they find those who influence their opinions most encouraging in their use of devices.

This group is the engine of rehabilitation programs: their commitment and positive attitude can serve as a model for others.

#### Liberated Adopters

Members of the second-largest group (32.4%) are open to using tools but are less active and, in some respects, more moderately engaged than enthusiastic users. The following attributes define this group: technology anxiety is the lowest, they rely most on family and friends for help, they have above-average device use, and they learn and use tools more easily than the average person.

This group is a stable base for the program: if given support and encouragement, they can easily become enthusiastic users.

#### Distrustful Reluctants

This is the smallest, but no less important, group of people (25/145, 17.2%) who are reticent and critical of rehabilitation technologies. This distrust is often due to anxiety, insecurity, or poor social support. They are the ones who enjoy using the devices less, are less attracted to trying new technologies, have above-average anxiety about using devices, are less likely to rely on family support, have more difficulty than average in learning to use new devices, and have the least belief in the role of device use in rehabilitation.

They are the group most in need of attention: personalized support, confidence-building, and mentoring can help them develop a more positive relationship with rehabilitation tools.

The cluster analysis was also performed on the questionnaire data collected in T2. We found that cluster blocks with practically identical characteristics can be distinguished, but their relative numbers of elements have changed. Among those who completed the program, the proportion of enthusiastic users increased from 50.3% to 61%, and the proportion of liberated adopters was almost the same (32.4% and 34.6%, respectively), while the proportion of distrustful reluctants decreased from 17.3% to 4.4%.

Due to the different cluster sizes and the varied distribution of participants, we cannot statistically say that different age groups behave differently. At the same time, the distribution of participants across clusters is not uniform: the group aged 60 years and older is concentrated in the “liberated adopters” cluster, whereas there are no participants aged 50 years and older in the “distrustful reluctants” cluster (although this cluster has the fewest members). Even after adjusting for unequal cluster sizes and the sample’s underlying age distribution, age remained associated with cluster membership, suggesting that age was a significant structuring variable of the clusters.

The association between sex and cluster membership was examined using a chi-square test of independence. The analysis indicated that sex distribution did not differ significantly among clusters (chi-square test, *P*>.05), suggesting that cluster membership was independent of sex.

## Discussion

### Principal Findings

As the results show, EE in the T1 study has no direct effect on intention to use, only through PE. This seems surprising at first sight, because EE usually has a direct positive effect on BI in UTAUT2 models. Although Venkatesh et al [23] suggested that if BI is highly dependent on PE, it may “absorb” the effect of EE, this has not been quantified. Several studies have found a significant relationship between EE and PE [43,58,59], but in no case did this prevent both constructs from also impacting BI. However, Kim et al [60] observed no direct effect of EE on BI when they used a UTAUT model to examine the adoption of mHealth tools and found that EE only affected BI as a moderating variable of trust. The lack of a direct effect of EE may be due to the fact that the experience of ease of use (EE) is not important per se, but because it increases the perception of usefulness (PE). In telerehabilitation settings, patients are not experience-driven but rather goal-driven, that is, they place more emphasis on whether the technology actually helps their recovery than on how easy it is to use.

In addition, as a result of the study in T1, although HM is included among the influencing constructs in UTAUT2, we did not measure its direct effect on intention to use (BI). Instead, HM moderates only through PE, that is, an enjoyable experience increases willingness to use only if it also positively influences perceived usefulness. Similar results are reported in the mHealth adoption study by Schretzlmaier et al [35], where the influence of PE and fear of illness on BI is clear, while the effect of HM is only conditional. The moderating effect of HM has also been demonstrated by Sitar-Tăut [61], who, in an m-learning setting, found that HM strongly influences PE within the HM-PE-BI sequence.

In addition, the analysis of the T1 study yielded the surprising result that trust has a negative effect on BI. This means that the more users trust the system, the less likely they are to want to use it. This seems to be a logical contradiction, as one would expect trust to have a positive effect on willingness to use, which is confirmed by several previous studies [34,35]. However, this phenomenon is consistent with a kind of safe bubble effect described in the literature [62], whereby users, due to excessive technological trust, feel less motivated to actively use technologies that require more interaction. Excessive technological confidence reduces user participation, and when confidence in the system exceeds perceived need, the active use of technology may even be abandoned [63]. In our case, this may have been reinforced by the technology’s actual trial, as data collection for the prerehabilitation analysis was conducted at the end of the preparation week, by which time, patients had learned to use the required tools in practice. In contrast, in T2, we found that the direct effect of trust on BI had disappeared, remaining relevant in the model only as a moderator. The experience gained during use and active participation in the operation is already reflected in the PE, resulting in a PE dominance in T2.

The analysis of the data recorded in T2 shows that the direct influence of SI, voluntariness, and safety on BI has also disappeared by the end of the telerehabilitation process. SI plays an important role in initiating device use, but later, this driving force is replaced by utility and effectiveness, embodied in PE. By examining the development of long-term device use habits, Peng et al [64] also found that SI before use is important, but that later regular use is no longer fueled by SI. The disappearance of voluntariness has no antecedent in the literature, as it is not included in the UTAUT2 model; it was reintroduced only under possible medical and eminence-based pressures, and its validity was confirmed by the T1 analysis. Sharif Abbasi et al [65], using the UTAUT model, found a result similar to ours when examining internet adoption over a longer period: while in T1, the effect of voluntariness on BI was measurable, in T2, it no longer remained significant, as experience with use increased. The disappearance of the effect of safety on BI can be explained by the fact that during device use, it becomes clear that the devices are secure and the data are safe, making this factor implicit in the patient’s behavior.

Of the constructs that directly influence technology use intention, only 2 remain: PE and FC, which appear in both the T1 and T2 models. While in the previous measurement, FC is by far the strongest (0.366) and PE is almost the weakest (0.232) direct effect on BI, in the latter measurement, FC’s influence is significantly weaker (0.258). One of the most important extensions of UTAUT2 was the introduction of user experience as a moderator on the constructs, including FC [23]. It was argued that, over time, as the routine of using the technology increases, FC becomes less crucial, sometimes even acting as a pullback force. By comparing the 2 datasets, we have now been able to provide empirical support for this hypothesis: the experience of using the tool for 3 months has led to a significant reduction in the impact of FC and a corresponding increase in the dominance of PE, which has “absorbed” the impact of SI, trust, and safety as well.

While clustering approaches commonly used in telemedicine are typically based on static categories such as socioeconomic status [66] or health literacy [67], we investigated how patients behave in a technological environment, what barriers they experience during use, and what motivational dynamics characterize them. The 3 groups explored represent different levels of technological engagement. This concept is more complex than technology acceptance because it includes emotional involvement (enjoyment or anxiety), motivation and volunteering, openness to novelty, willingness to learn, and self-efficacy, as well as the role of social support in technology use.

The cluster analysis of questionnaires collected before and after the telerehabilitation program shows that the program has a significant impact on patients’ intention to use. The relative share of enthusiastic users increased by about 20%, while the share of distrustful reluctants decreased to a quarter. Our results show that 2 factors are crucial for willingness to use: on the one hand, a meaningful trial of the technology (not just a demonstration, but an actual data collection simulating real-life situations at home) is the best way to dispel fears and anxieties about it, that is, a project where participants in a rehabilitation program can try out the technologies for a longer time during the preparation period can significantly contribute to their acceptance and willingness to use. On the other hand, the supportive and motivating role of the immediate environment is crucial; that is, where the family is indifferent or unsupportive, it is worthwhile to motivate family members with targeted communication during the preparation period of the telerehabilitation program.

### Conclusions

Our study has shown that the enabling environment is a key determinant of technology adoption in a telerehabilitation program. Consequently, when developing such a program, the focus should be on creating user-friendly interfaces, clearly communicating the benefits of technology and its relationship with other devices, and developing patients’ technological competencies. For patients more experienced with the technologies, PE will be the determining factor; if patients can experience the benefits of the technology in action during the preparation for the telerehabilitation program (training in the technologies and data delivery), their engagement will be stronger. If the impact of technological competency is to remain strong throughout the duration of a telerehabilitation program, it makes sense to adopt an adaptive strategy, that is, to measure increases in competency and reduce support intensity accordingly. This is both a way to signal user autonomy and to avoid a sense of excessive control. The cluster analysis also confirms that the key factor in the success of such a program is the tailored support and communication to the specific characteristics of each group, thereby strengthening their long-term commitment to participation. Indeed, the groups identified are not merely descriptive categories but operational profiles directly linked to the practical challenges of telerehabilitation. This functional approach, based on behavioral patterns, allows interventions to be personalized not according to assumed social characteristics but to real technology use needs. In clinical practice, using this, efforts should be made to identify clusters before initiating telerehabilitation. The next task is to develop personalized coaching techniques for each identified cluster and then examine whether attitudes toward technology use and the effectiveness of the entire telerehabilitation process can be improved through personalized interventions. We have already initiated developments in this direction, incorporating artificial intelligence support.

### Limitations

A limiting factor could be that, assuming a lack of technological experience, we omitted this from the model, even though there were certainly patients who possess such experience. We also did not assess whether the patients had participated in a similar rehabilitation program before or how this influenced their current attitudes. As the study was designed to compare intentions to use, actual use may have been influenced by factors we were unable to fully account for. With a larger sample size, it would also be possible to explain the behavioral differences observed across clusters using structural equation modeling analysis, which could be an exciting direction for future research.

## Appendix

Multimedia Appendix 1 Path analysis data for the preparatory week (T1) and the follow-up visit after the closing (T2).

## Abbreviations

BI: behavioral intention
CR: composite reliability
EE: effort expectancy
FC: facilitating condition
HM: hedonic motivation
HP: health care professional
mHealth: mobile health
OP: openness to innovation
PE: performance expectancy
SI: social influence
TAM: technology acceptance model
UTAUT: unified theory of acceptance and use of technology
UTAUT2: unified theory of acceptance and use of technology 2

## Data Availability

The datasets generated during this study are available in the Mendeley Data repository [68].
