# Supplementary material for: Role of Technology Acceptance in the Telerehabilitation of Patients With Metabolic Syndrome: Longitudinal Study
Source: JMIR Rehabil Assist Technol. 2026 May 14;13:e82161. doi: 10.2196/82161 (PMC13219986; doi:10.2196/82161)
Supplement: Multimedia Appendix 1 [file rehab_v13i1e82161_app1.docx]

**Table S1.** Path analysis data for the survey of the preparatory week (T1).

| **Path** | **Original sample** | **Sample mean** | **STDEV** | **t-statistics** | ***P* values** |
| --- | --- | --- | --- | --- | --- |
| AX - EE | -0.199 | -0.201 | 0.098 | 2.030 | .042 |
| AX - OP | -0.281 | -0.287 | 0.094 | 2.982 | .003 |
| AX - SA | -0.2 | -0.205 | 0.08 | 2.495 | .013 |
| EE - PE | 0.475 | 0.462 | 0.12 | 3.966 | 0 |
| FC - BI | 0.366 | 0.329 | 0.112 | 3.262 | .001 |
| HM - PE | 0.243 | 0.254 | 0.084 | 2.882 | .004 |
| HO - VO | 0.276 | 0.286 | 0.091 | 3.027 | .002 |
| HP - EE | 0.183 | 0.193 | 0.082 | 2.236 | .025 |
| HP - HO | 0.425 | 0.427 | 0.122 | 3.489 | 0 |
| HP - TR | 0.239 | 0.238 | 0.079 | 3.006 | .003 |
| OP - EE | 0.442 | 0.436 | 0.095 | 4.658 | 0 |
| PE - BI | 0.232 | 0.224 | 0.093 | 2.504 | .012 |
| SA - BI | 0.25 | 0.27 | 0.119 | 2.105 | .035 |
| SA - TR | 0.607 | 0.605 | 0.067 | 9.064 | 0 |
| SI - BI | 0.241 | 0.235 | 0.101 | 2.382 | .017 |
| TR - BI | -0.253 | -0.241 | 0.076 | 3.344 | .001 |
| VO - BI | 0.196 | 0.206 | 0.089 | 2.211 | .027 |

**Table S2.** Path analysis data for the survey at the follow-up visit after the closing (T2).

| **Path** | **Original sample** | **Sample mean** | **STDEV** | **t-statistics** | ***P* values** |
| --- | --- | --- | --- | --- | --- |
| AX - EE | -0.53 | -0.534 | 0.121 | 4.373 | 0 |
| AX - OP | -0.416 | -0.428 | 0.093 | 4.47 | 0 |
| AX - SA | -0.462 | -0.475 | 0.102 | 4.509 | 0 |
| EE - PE | 0.315 | 0.307 | 0.13 | 2.428 | .015 |
| FC - BI | 0.266 | 0.268 | 0.108 | 2.468 | .014 |
| HM - PE | 0.334 | 0.338 | 0.11 | 3.025 | .003 |
| HP - EE | 0.283 | 0.288 | 0.083 | 3.403 | .001 |
| HP - TR | 0.268 | 0.29 | 0.094 | 2.856 | .004 |
| PE - BI | 0.403 | 0.413 | 0.097 | 4.15 | 0 |
| SA - TR | 0.411 | 0.42 | 0.105 | 3.925 | 0 |
